# A life history model of the ecological and evolutionary dynamics of polyaneuploid cancer cells

**DOI:** 10.1038/s41598-022-18137-4

**Published:** 2022-08-12

**Authors:** Anuraag Bukkuri, Kenneth J. Pienta, Robert H. Austin, Emma U. Hammarlund, Sarah R. Amend, Joel S. Brown

**Affiliations:** 1 Cancer Biology and Evolution Program, Department of Integrated Mathematical Oncology, Moffitt Cancer Center, Tampa, USA; 2 The Brady Urological Institute, Johns Hopkins School of Medicine, Baltimore, USA; 3 Department of Physics, Princeton University, Princeton, USA; 4 Nordic Center for Earth Evolution, University of Southern Denmark and Translational Cancer Research, Department of Laboratory Medicine, Lund University, Lund, Sweden

**Keywords:** Population dynamics, Theoretical ecology, Evolutionary ecology, Ecological modelling, Evolutionary theory

## Abstract

Therapeutic resistance is one of the main reasons for treatment failure in cancer patients. The polyaneuploid cancer cell (PACC) state has been shown to promote resistance by providing a refuge for cancer cells from the effects of therapy and by helping them adapt to a variety of environmental stressors. This state is the result of aneuploid cancer cells undergoing whole genome doubling and skipping mitosis, cytokinesis, or both. In this paper, we create a novel mathematical framework for modeling the eco-evolutionary dynamics of state-structured populations and use this framework to construct a model of cancer populations with an aneuploid and a PACC state. Using in silico simulations, we explore how the PACC state allows cancer cells to (1) survive extreme environmental conditions by exiting the cell cycle after S phase and protecting genomic material and (2) aid in adaptation to environmental stressors by increasing the cancer cell’s ability to generate heritable variation (evolvability) through the increase in genomic content that accompanies polyploidization. In doing so, we demonstrate the ability of the PACC state to allow cancer cells to persist under therapy and evolve therapeutic resistance. By eliminating cells in the PACC state through appropriately-timed PACC-targeted therapies, we show how we can prevent the emergence of resistance and promote cancer eradication.

## Introduction

The emergence of resistance is a primary cause of treatment failure in cancer patients^1,2^. Fundamentally, therapeutic resistance arises because cancer cells evolve^3–7^. Their rate of evolution is the product of heritable variation (evolvability)^8–11^ and the force of selection, as imposed by therapy for instance^12–15^. The predominant view in cancer biology suggests that cancer cells, fueled by genomic instability, produce mutant progeny in the face of therapy^16^. Eventually, a resistant subpopulation of these cells will proliferate and render the cancer therapy ineffective. Recent evidence suggests another pathway to resistance: a poly-aneuploid cell state that cancer cells can enter during times of stress to avoid the effects of and adapt to extreme environmental conditions^17^. These enlarged cells have been noticed in many tumor types since the mid-19th century, but have largely been ignored as irreversibly senescent or destined for mitotic catastrophe^18^. One major reason they have not received much attention is the difficulty in experimentally measuring PACC populations. Currently, standard measures of cell response to therapy (e.g., dose response curves) cannot capture these rare cells that survive treatment as they fall below the limits of detection^19^. Furthermore, there are no biomarkers to detect PACC populations and flow cytometry methods, due to their long assay times and adverse effects on cell viability, are impractical^20^. Currently, the primary way to view or measure these cells is directly, through microscopy of in vitro cultures. Observing a cancer cell population upon induction of therapy reveals an increase in PACC frequency and number. At baseline, there are hardly any PACCs in the population; however, shortly after treatment with docetaxel, PACCs dominate the population^20^.

PACCs are formed when aneuploid cancer cells bypass mitosis, cytokinesis, or both and undergo whole genome doubling via cell fusion, endocycling, or mitotic slippage. This cell state is characterized by (1) an exit of the cell cycle after S phase to pause cell division^21^, allowing PACCs to endure stressful environments by avoiding DNA damage and programmed cell death, and (2) an increase in genomic content through polyploidization, promoting a greater generation of heritable variation that can be dispensed to their aneuploid 2N+ progeny upon depolyploidization via neosis or multipolar cell division^22–25^. This leads to the rapid generation of heritably resistant cells. Thus, the PACC state serves as a mechanism through which cancer populations can modulate their rate of evolution in response to their condition.

Understanding the ecological and evolutionary dynamics of PACC formation and depolyploidization is critical if we are to understand how resistance emerges and how it can effectively be prevented. To this end, we create an evolutionary game theoretic model^12,15^ of a cancer population with 2N+ and PACC states. This model allows us to explore two leading hypotheses regarding the role of the PACC state in promoting resistance: the non-proliferative hypothesis^26–28^, which holds that the PACC state provides cancer cells with a higher survivorship during times of stress by pausing cell division and protecting genomic material, and the evolutionary triage hypothesis^29–31^, which contends that the PACC state increases the generation of heritable variation in 2N+ progeny, accelerating the rate of evolution of cancer cells to therapy. Under these hypotheses, we simulate cancer cell population (ecological) and resistance strategy (evolutionary) dynamics under no therapy, continuous treatment, and intermittent therapy. We compare these results to those assuming a single 2N+ state in the population and show how the PACC state allows cancer cells to persist under therapy and/or evolve resistance to the stressor. Finally, we explore the efficacy of PACC-targeted therapies and demonstrate that, by administering therapy at appropriate times, we can prevent the emergence of resistance and promote cancer eradication.

## Model construction

To model the ecological (population) and evolutionary (resistance strategy) dynamics of a cancer cell population with 2N+ and PACC states, we use an evolutionary game theoretic approach called *G* functions^12^. The core of this framework is the *G* function that provides the fitness, or per capita growth rate, of an individual as a function of its strategy (*v*) and the strategies and population densities of other agents in the population ($\mathbf {u}$ and $\mathbf {x}$, respectively). This inherently game-theoretic formulation is important to consider as it allows us to capture not only the competition among cancer cells, but also the game between the cancer and the treatment administered by the physician. The population dynamics of the cancer cells is given by the product of the *G* function and the current population size:

1 $$\begin{aligned} \frac{dx}{dt} = xG(v,{\textbf {u}},{\textbf {x}}) \end{aligned}$$

By Fisher’s fundamental theorem of natural selection^14,32–34^, the strategy dynamics can be derived as the product of heritable variation (evolvability) and the selection gradient (e.g., as induced by therapy):

2 $$\begin{aligned} \frac{dv}{dt} = k\frac{dG}{dv} \end{aligned}$$

where *k* represents the trait’s evolvability and $\frac{dG}{dv}$ is the selection gradient, capturing how a perturbation in trait value impacts fitness.

To construct a *G* function model of the eco-evolutionary dynamics of state-structured cancer cell populations, we first construct a model of the underlying ecological dynamics of the system and determine how the resistance strategy impacts these dynamics. From this model, we then derive our *G* function and compute the evolutionary dynamics. This gives us a complete eco-evolutionary model, which we use to simulate and explore various biological questions.

### Ecological dynamics

First, we construct the ecological portion of the model. We let cells in the 2N+ state grow in a logistic fashion, with their growth equally inhibited in a density-dependent fashion by cells in the 2N+ and PACC state, until they reach their carrying capacity. Since cells cannot divide within the PACC state (rather, division requires depolyploidization into the 2N+ state), cells in the PACC state will have no natural growth rate. The effects of therapy are included via a death due to drug term for cells in the 2N+ state. We employed a Michaelis–Menten functional form for this term in which death due to drug depends on therapeutic dosage, a baseline level of resistance, and the resistance strategy^12,35–37^. Namely, as the cancer cell population evolves resistance to the therapy, *v* increases and mortality due to therapy decreases. We make two critical assumptions: the lack of strategy bounds and the absence of a cost of resistance^12^. These assumptions allow for an infinite improvement model under which cancer cells can theoretically evolve enough resistance to avoid the effects of therapy altogether. We assume that cells in the PACC state are fully resistant to therapy^20,38,39^. Thus, we do not include a death due to drug term in the PACC equation.

We allow for two different types of transitions from the 2N+ state to the PACC state: obligate (constant) and facultative (condition-dependent). Obligate transitions are supported experimentally from the baseline level of PACCs that exist in nearly all cancer cell populations^20^ and are incorporated into the model by a constant transition rate from the 2N+ to the PACC state. Facultative transitions are noticed by the increase in PACC number and frequency upon administration of therapy^40^ and are included in the model as proportional to the death due to therapy term. We also include a probability of successful poly-aneuploid transition parameter in each of these transitions to capture the fact that some transitions fail due to processes such as mitotic catastrophe. Finally, we assume a constant transition rate from the PACC state to the 2N+ state. Putting these components together, we arrive at a baseline model for PACC ecology in Eq. (3).

3 $$\begin{aligned} \frac{dx_1}{dt}&= \underbrace{rx_1\Big (\frac{K-x_1-x_2}{K}\Big )}_\text {Logistic Growth} \overbrace{-\gamma x_1}^\text {Obligate to PACC}\underbrace{-x_1\frac{m}{\lambda +bv}}_ \text {Drug-Induced Death}\overbrace{-c_{21}x_1\frac{m}{\lambda +bv}}^ \text {Facultative to PACC}\underbrace{+2c_{12}x_2}_\text {From PACC} \nonumber \\ \frac{dx_2}{dt}&= \underbrace{\gamma \zeta x_1}_\text {Obligate from 2N+}\overbrace{+c_{21}\zeta x_1\frac{m}{\lambda +bv}}^\text {Facultative from 2N+}\underbrace{-c_{12} x_2}_\text {To 2N+} \end{aligned}$$

The interpretations of each of these parameter values and baseline levels used in our simulations can be found in Table 1. Parameter values were chosen to be biologically plausible, numerically convenient for simulation purposes, and to clearly show differences among the various hypotheses on how PACCs contribute to therapeutic resistance. However, it is worth noting that many of these parameter values can qualitatively alter these results. For example, a higher growth rate, lower severity of therapy, or higher evolvability (as will be discussed in the upcoming section) can all dramatically improve the chances of the cancer surviving. Note that inherent in this model are the assumptions of a continuous quantitative trait that tracks the resistance profile to drug, homogeneity within the 2N+ and PACC states (i.e., all cells within a state have the same vital rates and parameter values), full resistance of PACCs to therapy, no natural background death rate, and pre-existence of an aneuploid cancer cell population. Despite these simplifying assumptions, our model will allow us to investigate hypotheses surrounding PACC formation, examine how PACCs contribute to therapeutic resistance, and how this resistance can be prevented with administration of PACC-targeted therapies.

**Table 1:** PACC parameter definitions and values used in simulations.

Parameter	Interpretation	Value
*r*	Intrinsic growth rate	0.6
*K*	Carrying capacity	100
$\gamma$	Obligate transition rate	0.02
*m*	Drug dosage	1
$\lambda$	Baseline level of resistance	1
*b*	Efficacy of evolving a resistance strategy	1
$c_{21}$	Facultative transition scaling rate	0.7
$c_{12}$	PACC to 2N+ Transition rate	0.2
$\zeta$	Successful poly-aneuploid transition rate	0.7
*v*	Drug resistance	$[0,\infty )$

### Evolutionary dynamics

With the model of cancer cell ecology, we now derive the relevant *G* function and thereby obtain the evolutionary dynamics. Before doing this, it’s worth noting that we hypothesize that PACCs serve as a distinct *life history state* in the life cycle of cancer cells, not as a distinct cell type. In other words, cancer cells can fluidy enter and exit the PACC state, in a facultative manner, depending on environmental conditions. Distinct life history states are ubiquitous in ecology, from the larvae and adult fly to the polyp and adult jellyfish. Though organisms in each of these stages are part of the same species, each stage is characterized by different strategies for survival and different responses to the environment. Due to this, we cannot directly read off the *G* functions from the 2N+ and PACC equations in Eq. (3). Instead of having a separate *G* function for each state, since cells in both 2N+ and PACC states are part of the same species, we must derive a unifying *G* function that incorporates both in a life history-enlightened manner. To do this, we follow an approach introduced in our recent paper^41^ where we represent our model as a population projection matrix (PPM)^42–44^ and use the spectral bound of the matrix as a measure of fitness, as it controls the long-term (asymptotic) growth rate of the population. Namely, our PPM takes the following form:

$$\begin{aligned} A = \begin{bmatrix} r\left( \frac{K-x_1-x_2}{K}\right) -\gamma -\left( 1+c_{21}\right) \frac{m}{\lambda +bv} &{} 2c_{12}\\[12pt] \zeta \left( \gamma +c_{21}\frac{m}{\lambda +bv}\right) &{} -c_{12} \end{bmatrix} \end{aligned}$$

Then, we can set $G=\rho (A)$ where $\rho (A)$ represents the spectral bound of the PPM. Thus, we can capture the evolutionary dynamics of resistance as follows:

4 $$\begin{aligned} \frac{dv}{dt} = k\frac{d\rho (A)}{dv} \end{aligned}$$

Note that under this formulation, we can explore traditional models of cancer evolution that assume a single 2N+ state in the population by removing the PACC state entirely. This leads to a PPM with a single entry, whose spectral bound is directly this element (*G* function) itself. We can explore the hypothesis that PACCs allow cells to“hibernate”and provide a refuge from stressful conditions by setting the transition from PACCs to 2N+ cells (bottom right element of the PPM) to 0 when under therapy. Finally, we can investigate the hypothesis that PACCs accelerate the evolution of resistance by letting the evolvability be a weighted average of PACC and 2N+ frequencies in the population, with PACCs contributing to a greater evolvability. It is worth noting that since we use the *G* function framework to model the evolutionary dynamics of the cancer cell population, we obviate the need to explicitly define sensitive and resistant 2N+ subpopulations. Instead, we can use a mean-field approximation of the evolutionary dynamics of a point cloud distribution, functionally treating cells within 2N+ and PACC states as homogeneous and tracking the mean trait over time.

Now that we have equations that allow us to couple ecological and evolutionary dynamics, we can begin investigating questions surrounding 2N+/PACC dynamics under therapy. To produce all the following simulations, we solve the respective system of ODEs numerically using Python’s odeint library.

## Cancer eco-evolutionary dynamics

### Single-state model

Traditionally, resistance in cancer models is assumed to occur through a single state in the population. This case, analogous to the standard tumor heterogeneity hypothesis^16^, assumes that resistance is the result of a selective expansion of genetically or epigenetically distinct subpopulations^45^. These subpopulations could, due to inherent tumor heterogeneity, have pre-existing resistance to the applied therapy or could acquire resistance mutations by random chance, fueled by aneuploidy and genomic instability^38,46,47^. The evolution of resistance to each therapy requires a different set of mutations to arise, stochastically, by at least one cell in the tumor. Models that operate under this hypothesis do not account for other states cells can enter to increase their evolution of resistance or survive under therapy-resistance is developed solely by cells accruing rare beneficial mutations that improve their fitness, allowing them to expand in the population.

To simulate this hypothesis, we exclude the PACC state from our model entirely. Thus, only a single 2N+ state exists in the population with the following ecological and evolutionary dynamics, derived from Eq. (3) as:

5 $$\begin{aligned} \frac{dx}{dt}&= \underbrace{rx\left( \frac{K-x}{K}\right) }_\text {Logistic Growth} \overbrace{-x\frac{m}{\lambda +bv}}^\text {Drug-Induced Death} \nonumber \\ \frac{dv}{dt}&= k\frac{bm}{(bv+l)^2} \end{aligned}$$

**Figure 1:**
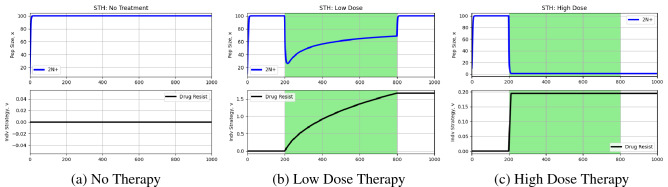
Simulations under standard tumor heterogeneity hypothesis. The black curve captures the evolutionary dynamics of resistance White and green backgrounds reflect periods without and periods with treatment, respectively. The rate of evolution under the standard tumor heterogeneity hypothesis is enough to stave off extinction and undergo evolutionary rescue when faced with a low dose therapy. However, under high dose therapy, the cancer cells cannot evolve fast enough to remain extant.

Now, we are ready to simulate cancer cell dynamics under the tumor heterogeneity hypothesis. We simulate three cases: no therapy ($m=0$), a low dose of therapy ($m=0.5$), and a high dose of therapy ($m=1$). We administer therapy continuously from time step 200 until time step 800. The results from these simulations can be seen in Fig. 1.

As expected, when no treatment is administered (Fig. 1), no resistance develops and the cancer cells reach their carrying capacity at 100. When a low dose of therapy is given (Fig. 1), the cancer cells are able to undergo evolutionary rescue and evolve a resistance strategy fast enough to stave off extinction and reach an equilibrium at carrying capacity. However, under high dose therapy (Fig. 1), the cells are not able to evolve resistance fast enough to remain extant—they are eradicated shortly after therapy is administered.

### Non-proliferation

Next, let’s look at one of the two leading hypotheses on how PACCs contribute to resistance: the non-proliferative (NP) hypothesis. The NP theory is analogous to a hibernation or bet-hedging strategy where species enter a dormant state in response to harsh environmental conditions, lowering their metabolism and activity^48^. Once the environment improves, these species can awake from hibernation and proceed with their usual activities^49,50^. In cancer, PACCs may similarly serve as a (reversible) quiescent state that cancer cells can enter when faced with a novel stressor^26–28^. The key role of PACCs under this hypothesis is to allow cancer cells to *survive* during periods of extreme stress by exiting the cell cycle and protecting their genome^51,52^. Once the stressor is removed or conditions otherwise improve, the PACCs can undergo depolyploidization, producing aneuploid progeny that can continue proliferating. Note that under this hypothesis, no mutations are required to develop resistance—survival is sufficient. To simulate this, we set the transition from the PACC to 2N+ state to zero if death due to therapy reaches or surpasses a certain threshold (set at 1 in this case).

**Figure 2:**
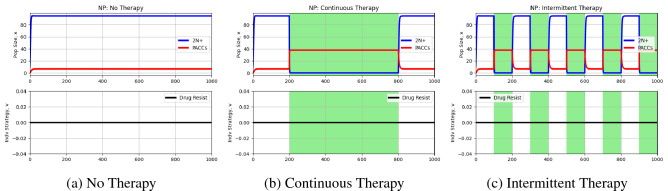
Simulations under non-proliferation hypothesis. Blue and red curves depict 2N+ and PACC population dynamics, respectively, and the black curve captures the evolutionary dynamics of resistance. White and green backgrounds reflect periods without and periods with treatment, respectively. During times of treatment, the population is able to persist by entirely existing in the PACC state. During drug holidays, the cells switch to a predominantly 2N+ state. Since PACCs are not killed by therapy, the selection gradient with respect to drug resistance is zero and hence, resistance does not evolve.

We simulate three cases: no therapy, continuous therapy ($m=1$) from time step 200 to time step 800, and intermittent therapy ($m=1$) under which therapy is turned on and off every 100 time steps. The results from these simulations can be seen in Fig. 2.

When no treatment is administered (Fig. 2), a baseline level of PACCs exist in the population and there is no development of resistance. In both continuous (Fig. 2) and intermittent therapy (Fig. 2) cases, during times of drug holidays, the majority of cells are in the 2N+ state, with a baseline level remaining in the PACC state. When under therapy, all cells in the population switch to the PACC state to avoid the effects of the drug. Since no death due to drug occurs in the PACC state, there is no selective pressure to evolve resistance. Thus, we observe a temporary state of resistance in the population when therapy is administered, which returns back to a sensitive state when the drug is removed.

### Evolutionary triage

**Figure 3:**
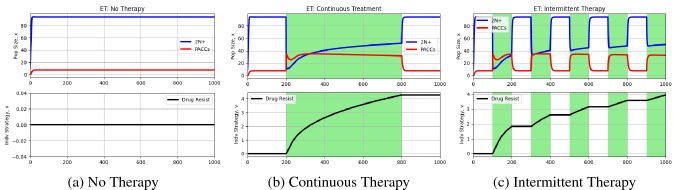
Simulations under evolutionary triage hypothesis. Blue and red curves depict 2N+ and PACC population dynamics, respectively, and the black curve captures the evolutionary dynamics of resistance. White and green backgrounds reflect periods without and periods with treatment, respectively. When therapy is administered, a large portion of the population switches from the 2N+ to the PACC state, avoiding the effects of therapy and increasing evolvability. As resistance emerges, the frequency of cells in the PACC state decreases.

Now, let’s turn to another hypothesis for how PACCs promote resistance: evolutionary triage (ET). The ET theory suggests that the increase in genomic material associated with the PACC state not only promotes genomic stability, preventing apoptosis, but also creates greater heritable variation in their progeny^29–31^. The key role of PACCs here is to provide greater heritable variation in progeny, speeding up the rate of evolution and allowing cancer cells to more readily adapt to novel stressors. In other words, natural selection acts upon the large diversity of progeny generated by the PACCs with the fitter variants proliferating at the expense of the less fit ones. The larger the diversity in progeny (with respect to fitness), the more efficiently natural selection can act and the faster evolution can proceed. To simulate this, we let evolvability depend on the proportion of 2N+ cells and PACCs in the population, with PACCs contributing to a higher evolvability:

6 $$\begin{aligned} k = \frac{k_{1}x_1+k_{2}x_2}{x_1+x_2} \end{aligned}$$

where $k_1<k_2$. As before, we simulate three cases: no therapy, continuous therapy ($m=1$) from time step 200 to time step 800, and intermittent therapy ($m=1$) under which therapy is turned on and off every 100 time steps. The results from these simulations can be seen in Fig. 3.

Again, when no treatment is administered (Fig. 3), the population comprises of cells predominantly in the 2N+ state with a baseline frequency in the PACC state. Under continuous treatment (Fig. 3), the cancer cells become increasingly resistant as the simulation progresses. Due to this increasing resistance, over time, there is less of a need for heritable variation to be generated. As such, the PACC population gradually decreases after treatment administration. In the intermittent therapy case (Fig. 3), we see periods of treatment promoting evolution of the strategy towards higher resistance levels and periods without treatment maintaining evolutionary stasis. The population dynamics parallel these trends as well, with treatment periods increasing the PACC population while decreasing the 2N+ population and periods without treatment having the opposite effect. Note that over time, however, there is a general trend of a decreasing PACC population since the cancer cell population steadily gains resistance to the drug. It is worth noting that, under this hypothesis, the PACC state both provides refuge from therapy, dampening the selective pressure on the cells to develop resistance, as well as increasing the evolvability of the cancer cells, accelerating the evolution of resistance. These two sides contribute, in opposing directions, to the overall rate of evolution of resistance that we observe.

## PACC-targeted therapies

In the cases explored so far, the PACC state has allowed the cancer cell population to persist in the face of therapy, evolve resistance, or both. In order to prevent the emergence of resistance and improve long-term outcomes of patients, therapeutic strategies that target PACC populations is essential. We hypothesize that by eliminating cells in the PACC state, we can promote collapse of the tumor ecosystem and prevent relapse. To do this, drugs can be developed that target each PACC life history transition: preventing formation of PACCs, directly inducing death in PACCs, or preventing PACC depolyploidization. Once viable drugs are identified, it is imperative to determine how to best use them. To this end, we will simulate administration of a chemotherapeutic agent from time 200 to 800 in addition to a PACC-targeted therapy to promote cancer eradication. This PACC-targeted therapy will be administered before (time 150–200), during (time 200–250), or after (250–300) the start of chemotherapy. For each case, we will simulate 2N+ and PACC eco-evolutionary dynamics under both NP and ET hypotheses.

### Preventing PACC depolyploidization

One way to target cells in the PACC state is by preventing depolyplodization when these cells attempt to transition to the 2N+ state. Blocking asymmetric mitosis through prevention of centrosome clustering with a Kinesin family member C1 (KIFC1) inhibitor such as 2-(3-pyridylmethyl)-5-nitro-2-furamide is one possible way to do this^39,53–56^. To incorporate KIFC1 inhibitors into Eq. (3), we will modify the 2N+ from PACC transition term to be a decreasing function of drug dosage. The amended model can thus be seen below with $n=0.8$ representing the dosage of the KIFC1 inhibitor:

7 $$\begin{aligned} \frac{dx_1}{dt}&= \underbrace{rx_1\Big (\frac{K-x_1-x_2}{K}\Big )}_\text {Logistic Growth}\overbrace{-\gamma x_1}^\text {Obligate to PACC}\underbrace{-x_1\frac{m}{\lambda +bv}}_\text {Drug-Induced Death}\overbrace{-c_{21}x_1\frac{m}{\lambda +bv}}^\text {Facultative to PACC}\underbrace{+2c_{12}\left( 1-n\right) x_2}_\text {From PACC w/KIFC1 Inhibitor}\nonumber \\ \frac{dx_2}{dt}&= \underbrace{\gamma \zeta x_1}_\text {Obligate from 2N+}\overbrace{+c_{21}\zeta x_1\frac{m}{\lambda +bv}}^\text {Facultative from 2N+}\underbrace{-c_{12}x_2}_\text {To 2N+} \end{aligned}$$

**Figure 4:**
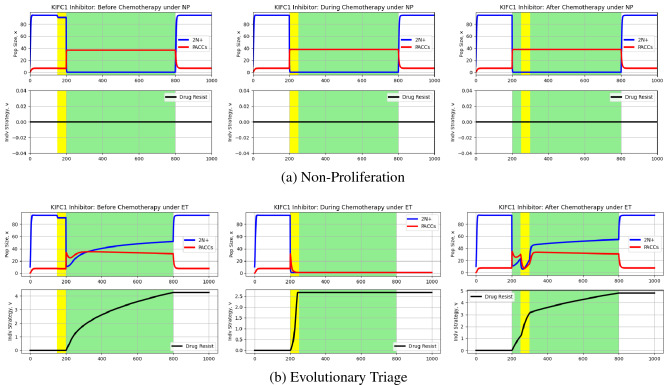
Effect of KIFC1 inhibitor on eco-evolutionary dynamics of cancer cell population. Blue and red curves depict 2N+ and PACC population dynamics, respectively, and the black curve captures the evolutionary dynamics of resistance. White and green backgrounds reflect periods without and periods with chemotherapy, respectively, and yellow backgrounds are periods with a KIFC1 inhibitor. Under ET, the KIFC1 inhibitor promoted extinction of the cancer cell population when administered at the same time as chemotherapy. Under NP, the KIFC1 inhibitor had little impact on 2N+ and PACC dynamics.

Using this model, we can now run simulations of 2N+ and PACC eco-evolutionary dynamics under chemotherapy and a KIFC1 inhibitor, as described earlier. The results of these simulations can be seen in Fig. 4.

First, let’s what happens under the NP hypothesis (Fig. 4). Since KIFC1 inhibitors only impact the depolyploidization of cells from the PACC to the 2N+ state, they have no impact on cellular dynamics when administered during or after the start of chemotherapy since no depolyploidization is occurring at this time. We notice a minute decrease in the 2N+ population when the KIFC1 inhibitor is given before the start of chemotherapy—this is due to the small fraction of cells that are undergoing an obligate transition from the the PACC to the 2N+ state.

Now, let’s turn our attention to the dynamics under the ET hypothesis (Fig. 4). When the KIFC1 inhibitor is given at the same time as chemotherapy, we notice that cells in both the 2N+ and PACC states are driven to extinction. Let’s consider how this happens. Upon administration of chemotherapy, there is a massive transition of cells from the 2N+ to PACC state, accompanied by the death cells in the 2N+ state experience due to therapy. Soon after, cells in the PACC state attempt to depolyploidize into the 2N+ state. This is blocked by the KIFC1 inhibitor that eliminates these cells. Since the transitioning PACCs can no longer contribute to the aneuploid population and not enough resistance has been achieved, the 2N+ population crashes. This removes the source for the PACC population, which simply declines to extinction as cells continue attempting to transition to the 2N+ state unsuccessfully. However, if the KIFC1 inhibitor is given 50 time steps after the administration of chemotherapy, the cancer population is not driven to extinction. Namely, the cancer cells have already gained some resistance and have a larger population size than immediately after therapy. Due to this, although we see a clear decrease in the 2N+ and PACC populations, the cancer is able to undergo evolutionary rescue and remain extant. Finally, as we saw in the NP case, administering the KIFC1 inhibitor before chemotherapy does little to impact population dynamics, making a minor dent in the 2N+ population due to inhibition of the obligate transition from the PACC to the 2N+ state.

### Preventing PACC formation

Preventing PACC formation, e.g., through the use of cyclin/CDK inhibitors, represents another promising way to target cells in the PACC state^57–59^. To incorporate cyclin/CDK inhibitors into Eq. (3), we will modify the PACC from 2N+ transition term to be a decreasing function of drug dosage. The revised model can be seen below with $n=0.8$ representing the dosage of the cyclin/CDK inhibitor:

8 $$\begin{aligned} \frac{dx_1}{dt}&= \underbrace{rx_1\Big (\frac{K-x_1-x_2}{K}\Big )}_\text {Logistic Growth}\overbrace{-\gamma x_1}^\text {Obligate to PACC}\underbrace{-x_1\frac{m}{\lambda +bv}}_\text {Drug-Induced Death}\overbrace{-c_{21}x_1\frac{m}{\lambda +bv}}^\text {Facultative to PACC}\underbrace{+2c_{12}x_2}_\text {From PACC}\nonumber \\ \frac{dx_2}{dt}&= \underbrace{\gamma \zeta x_1\left( 1-n\right) }_\text {Obligate from 2N+ with cyclin/CDK inhibitor}\overbrace{+c_{21}\zeta x_1\frac{m}{\lambda +bv}\left( 1-n\right) }^\text {Facultative from 2N+ with cyclin/CDK inhibitor}\underbrace{-c_{12}x_2}_\text {To 2N+} \end{aligned}$$

**Figure 5:**
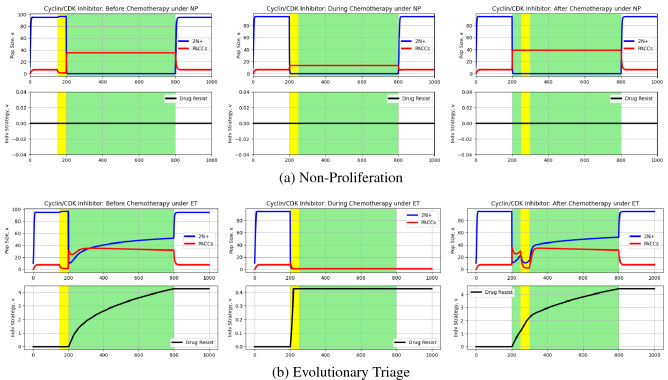
Effect of cyclin/CDK inhibitors on eco-evolutionary dynamics of cancer cell population. Blue and red curves depict 2N+ and PACC population dynamics, respectively, and the black curve captures the evolutionary dynamics of resistance. White and green backgrounds reflect periods without and periods with chemotherapy, respectively, and yellow backgrounds are periods with a Cyclin/CDK inhibitor. Under ET, the Cyclin/CDK inhibitor promoted extinction of the cancer cell population when administered at the same time as chemotherapy. Under NP, the Cyclin/CDK inhibitor was able to greatly reduce the PACC population when administered just before or at the same time as chemotherapy, but was ultimately unable to eradicate the cancer.

Using this model, we can now run simulations of 2N+ and PACC eco-evolutionary dynamics under chemotherapy and a cyclin/CDK inhibitor, as described earlier. The results of these simulations can be seen in Fig. 5.

Again, let’s consider the results under the NP hypothesis first (Fig. 5). Cyclin/CDK inhibitors impact the transition from the 2N+ state to the PACC state, preventing PACC formation. When given before chemotherapy, we see a drastic decrease in the PACC population (accompanied by a very minor increase in the 2N+ population). However, upon administration of therapy, cells in the 2N+ state are able to transition to the PACC state and avoid the effects of therapy. When the cyclin/CDK inhibitor is given at the same time as chemotherapy, cells in the 2N+ state are not able to effectively transition into the PACC state. However, the therapy is unable to eliminate the PACC population entirely and, after therapy is removed, the population returns to pre-treatment levels. The cyclin/CDK inhibitor is found to have no impact on dynamics when given after chemotherapy. This is because all cells are in the PACC state when the inhibitor is given. Thus, there is no transition from the 2N+ to PACC state occurring.

Next, let’s examine the eco-evolutionary dynamics under ET (Fig. 5). Just as occurred under NP, the cyclin/CDK inhibitor is effective at reducing the PACC population when given prior to the start of chemotherapy, but the population is still able to undergo evolutionary rescue once chemotherapy is commenced due to the large 2N+ population that serves as the source for new PACCs. However, when the inhibitor is given during chemotherapy, it proves to be incredibly effective. Namely, in inhibits the critical transition from 2N+ cells to PACCs upon administration of therapy, quickly leading to the eradication of both 2N+ and PACC population before hardly any resistance emerges. Similar to the KIFC1 inhibitor results, when cyclin/CDK inhibitor administration is delayed, it still proves to be highly effective at reducing PACC and 2N+ population sizes, but due to the existing resistance levels and population size, is not able to drive the cancer to extinction.

### Direct PACC killing

The final way we will consider to target PACCs is by directly killing cells that are in the PACC state. Recent evidence has shown that this may be possible by directed modulation of cellular metabolism by targeting lipid droplets^60^. To incorporate metabolism modulation into Eq. (3), we will include a direct PACC killing term. The updated model can then be seen below with $n=0.8$ representing the dosage of the metabolic modulator:

9 $$\begin{aligned} \frac{dx_1}{dt}&= \underbrace{rx_1\Big (\frac{K-x_1-x_2}{K}\Big )}_\text {Logistic Growth} \overbrace{-\gamma x_1}^\text {Obligate to PACC} \underbrace{-x_1\frac{m}{\lambda +bv}}_\text {Drug-Induced Death}\overbrace{-c_{21}x_1\frac{m}{\lambda +bv}}^\text {Facultative to PACC}\underbrace{+2c_{12}x_2}_\text {From PACC}\nonumber \\ \frac{dx_2}{dt}&= \underbrace{\gamma \zeta x_1}_\text {Obligate from 2N+}\overbrace{+c_{21}\zeta x_1\frac{m}{\lambda +bv}}^\text {Facultative from 2N+}\underbrace{-c_{12}x_2}_\text {To 2N+}\overbrace{-nx_2}^\text {Metabolism Modulation Death} \end{aligned}$$

**Figure 6:**
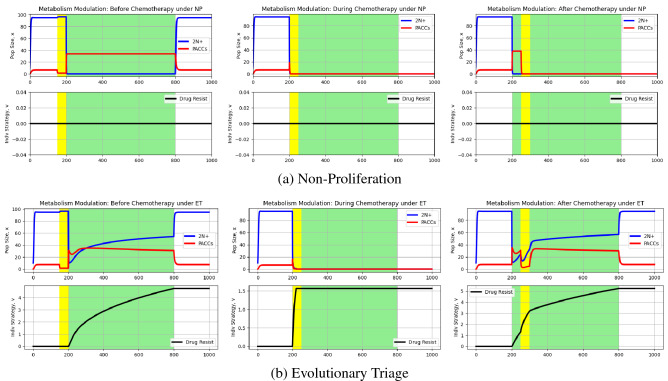
Effect of metabolism modulation on eco-evolutionary dynamics of cancer cell population. Blue and red curves depict 2N+ and PACC population dynamics, respectively, and the black curve captures the evolutionary dynamics of resistance. White and green backgrounds reflect periods without and periods with chemotherapy, respectively, and yellow backgrounds are periods with a metabolic drug. Under ET, the metabolic drug promoted extinction of the cancer cell population when administered at the same time as chemotherapy. Under NP, the metabolic drug was able to eradicate the cancer when administered at the same time as or 50 time steps after the start of chemotherapy.

Using this model, we can now run simulations of 2N+ and PACC eco-evolutionary dynamics under chemotherapy and a metabolism modulator, as described earlier. The results of these simulations can be seen in Fig. 6.

Let’s investigate the NP results first (Fig. 6). When the metabolism modulator is given before chemotherapy, just as in the cyclin/CDK case, the PACC population is dramatically reduced. However, once chemotherapy commences, cells in the 2N+ state rapidly transition to the PACC state, allowing the cancer to persist in the face of therapy. However, when the modulator is given during or after the start of chemotherapy, cancer eradication is possible. In a straightforward manner, the drug simply kills the PACC cells, thereby eradicating the tumor since no cells exist in the 2N+ state under therapy. After therapy is removed, there are no PACCs left to help repopulate the tumor.

Now, consider the dynamics under ET (Fig. 6). The results when the modulator is given before therapy are analogous to those under NP and under the cyclin/CDK inhibitor. When given during chemotherapy, cells in the 2N+ state dramatically attempt to transition to the PACC state, but this is to no avail as the metabolism modulator effectively kills the PACC cells. This leads to the eradication of both the PACC and 2N+ population as the cells cannot gain enough resistance to undergo evolutionary rescue without the PACC population. Finally, as in all the PACC-targeted therapies we considered, delaying administration of the metabolism modulator does dramatically decrease the PACC and 2N+ population, but not enough to result in extinction.

## Discussion

In this paper, we created an evolutionary game theoretic model, based on a novel life history *G* function approach, to examine the eco-evolutionary dynamics of a state-structured population with 2N+ and PACC states. We compared the results from a model that assumes a single state in the population (tumor cell heterogeneity) with models that also allow for a PACC state (non-proliferation and evolutionary triage). We simulated population and resistance strategy dynamics under the latter two hypotheses for continuous and intermittent therapy regimens to demonstrate the two ways PACCs can promote resistance: by allowing cancer cells to avoid the effects of therapy and by increasing the generation of heritable variation to accelerate the evolution of resistance. We then examined the efficacy of PACC-targeted therapies for cancer eradication. We specifically looked at therapies that target three key aspects in the life cycle of cancer cells: formation of the PACC state (with cyclin/CDK inhibitors), the stable PACC state (with metabolism modulation), and depolyploidization from the PACC state (with KIFC1 inhibitors). For each of these therapies, we found that administration of the PACC-targeted therapy at the same time as chemotherapy was most effective. Under KIFC1 and cyclin/CDK inhibitors, this caused eradication of the cancer under the ET hypothesis. Under metabolism modulators, administration of the PACC-targeted therapy caused cancer extinction under ET and NP when given at the start of chemotherapy and caused extinction under NP also when given after the start of chemotherapy.

Theoretically, our novel state-structured population modeling approach has wide implications for biology. There are many problems in which understanding and incorporating state-structure into modeling is critical to gain an understanding of the underlying ecological and evolutionary dynamics. For example, when modeling migration among habitats, immune cell polarization, or animal development, ignoring the inherent state-structure in the population forces the modeler to assume that organisms in each of these states are different species, attempting to maximize their own fitness. Though this assumption is permissible in a technical sense when dealing solely with ecological dynamics, as soon as evolutionary dynamics become relevant, the danger of this assumption becomes clear: evolution does not increase the fitness of organisms *within* particular states, but rather *across* states. Our state-structured modeling approach allows us to do just this: it provides a broadly applicable way to incorporate a quantitative, continuous, evolving trait across organisms in various states.

In the context of cancer, our work demonstrates the importance of including cell states in models of eco-evolutionary dynamics. Critically, it shows how the PACC state contributes to therapeutic resistance and stresses the importance of eliminating this keystone species via appropriately-timed PACC-targeted therapies to promote collapse of the tumor ecosystem. Several models already exist that attempt to include different cell states into population modeling, e.g., by having sensitive and resistant populations^61,62^. However, our model is distinguished from these in two critical ways. First, these models do not allow for tracking of a continuous, quantitative trait across states as ours does and treat sensitive and resistant states as different species, each maximizing their own fitness (frequency in the population). Secondly, in these models, each state or species is proliferative in their own right. However, in our model, although the 2N+ state is proliferative and can survive on their own, the PACCs are truly a non-proliferative life history state that requires 2N+ cells.

Since PACCs are implicated across many cancer types and stressors, we expect that these findings will apply across a broad range of cancers in a variety of therapeutic and environmental contexts. As the first modeling study to explicitly incorporate the PACC life history state, we hope that future work will build upon this model with more detailed and intricate aspects of PACC biology. As this study is theoretical, we acknowledge that further experimental work is needed to test and extend these results. However, it is clear that PACCs have the potential to be the source of therapeutic resistance in cancer and more effort must be dedicated to understand how this occurs and develop therapies to target this life history state.

## Data Availability

All data generated or analysed during this study are included in this published article.
